# Salticidae (Arachnida, Araneae) of Thailand: new species and records of *Epeus* Peckham & Peckham, 1886 and *Ptocasius* Simon, 1885

**DOI:** 10.7717/peerj.9352

**Published:** 2020-06-22

**Authors:** Barbara Maria Patoleta, Joanna Gardzińska, Marek Żabka

**Affiliations:** 1 Faculty of Exact and Natural Sciences, Siedlce University of Natural Sciences and Humanities, Siedlce, Poland; 2 Warsaw College of Engineering and Health, Warsaw, Poland

**Keywords:** Salticidae, Jumping spiders, Oriental region, Taxonomy, Distribution, Thailand, *Epeus*, *Ptocasius*

## Abstract

The study is based on new material from the collections of the Naturalis Biodiversity Centre in Leiden (RNHM) and the Hungarian Natural History Museum (HNHM) and addresses issues in two genera: *Epeus* Peckham & Peckham, 1886 and *Ptocasius* Simon, 1885 from Thailand. Both genera are of Asian/Indomalayan origin, the latter with a diversity hotspot in the subtropical valleys of the Himalayas. Based on morphological data, we propose three new species of *Epeus* (*Epeus daiqini* sp. nov. (♂♀), *Epeus pallidus* sp. nov. (♀), *Epeus szirakii* sp. nov. (♀)) and two new species of *Ptacasius* (*Ptocasius metzneri* sp. nov. (♂♀) and *Ptocasius sakaerat* sp. nov. (♀)). Additionally, we redescribed *E*. *tener* (Simon, 1877) and added photographs of morphological characters. The genus *Ptocasius* is redefined due to the inclusion of 37 species, previously included in *Yaginumaella* Prószyński, 1979. Relationships and distribution of both genera are discussed in reference to molecular, morphological and distributional data, published by other authors in recent years.

## Introduction

The list of jumping spiders from Thailand comprises 29 genera and 46 species (WSC, 2020), seven genera and 13 species being poorly documented and in need of verification (Żabka & Gardzińska, 2017). The comparison of Thailand salticid checklist with other countries of the region (126 species in Vietnam, 511 in China, 386 in Indonesia; Metzner, 2020) illustrates that the real numbers for Thailand are seriously underestimated and could reach several hundred species as is found in Vietnam (Żabka, 1985). In this study we describe five new species from two genera: *Epeus* (Peckham & Peckham, 1886) and *Ptocasius* (Simon, 1885), the latter being recorded in Thailand for the first time. Both genera are of Asiatic origin (Bodner & Maddison, 2012; Maddison, 2015), and are found in SE Asia, China and subtropical Himalayas (Jastrzębski, 2010). The Himalayas have become a biodiversity hotspot for *Ptocasius,* with a large number of closely related species, all of them previously described in *Yaginumaella* (Prószyński, 1979).

The goal of the paper is to describe new species of *Epeus* and *Ptocasius*, to redefine their diagnoses and to discuss the distribution of each genus.

## Material & Methods

**Figure 1:**
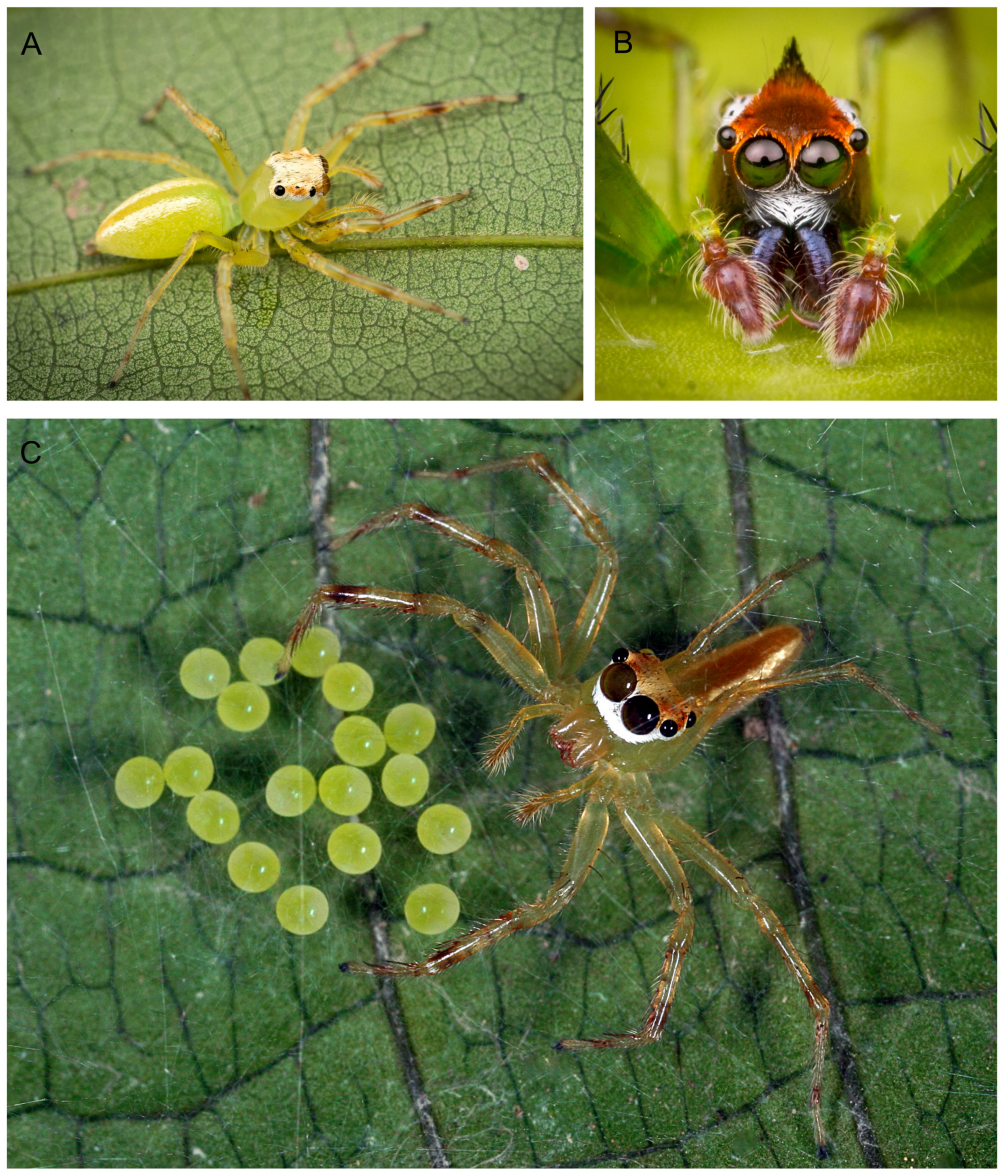
*Epeus sp*.—live specimens. (A) Dorso-lateral view; (B) frontal view (image credit: Joseph Kok Hong Koh); (C) female with eggs (image credit: David John Court).

**Figure 2:**
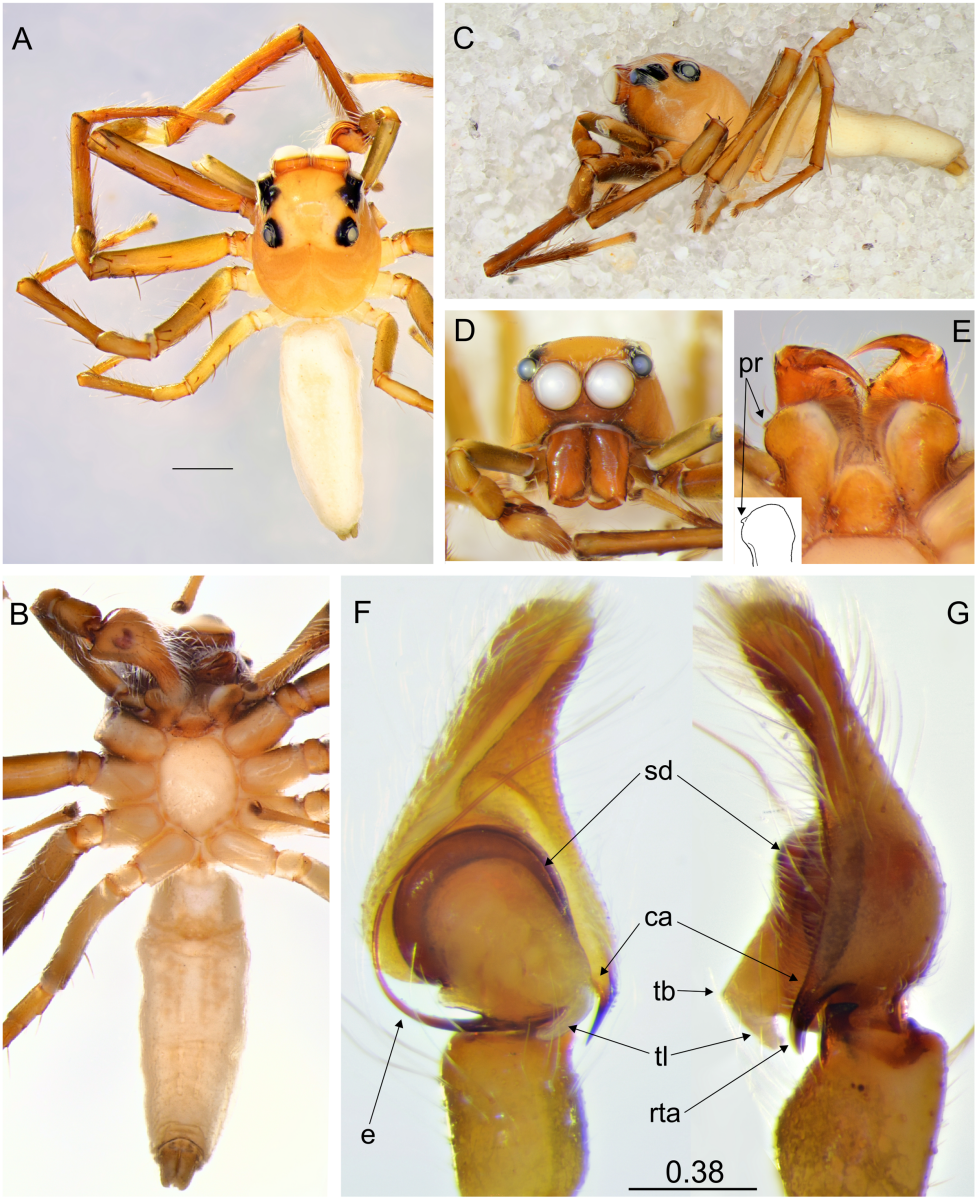
*Epeus tener*. (A) Dorsal view; (B) ventral view; (C) lateral view; (D) frontal view; (E) chelicerae, endites and labium (arrows indicate protuberance on endite); (F) right palp ventrally; (G) same, retrolaterally. (Abbreviations: ca: cymbial apophysis, e: embolus, pr: protuberance, rta: retrolateral tibial apophysis, sd: seminal duct, tb: tegular bump, tl: tegular lobe). Scale bar: 1 mm, F–G as in fig.

**Figure 3:**
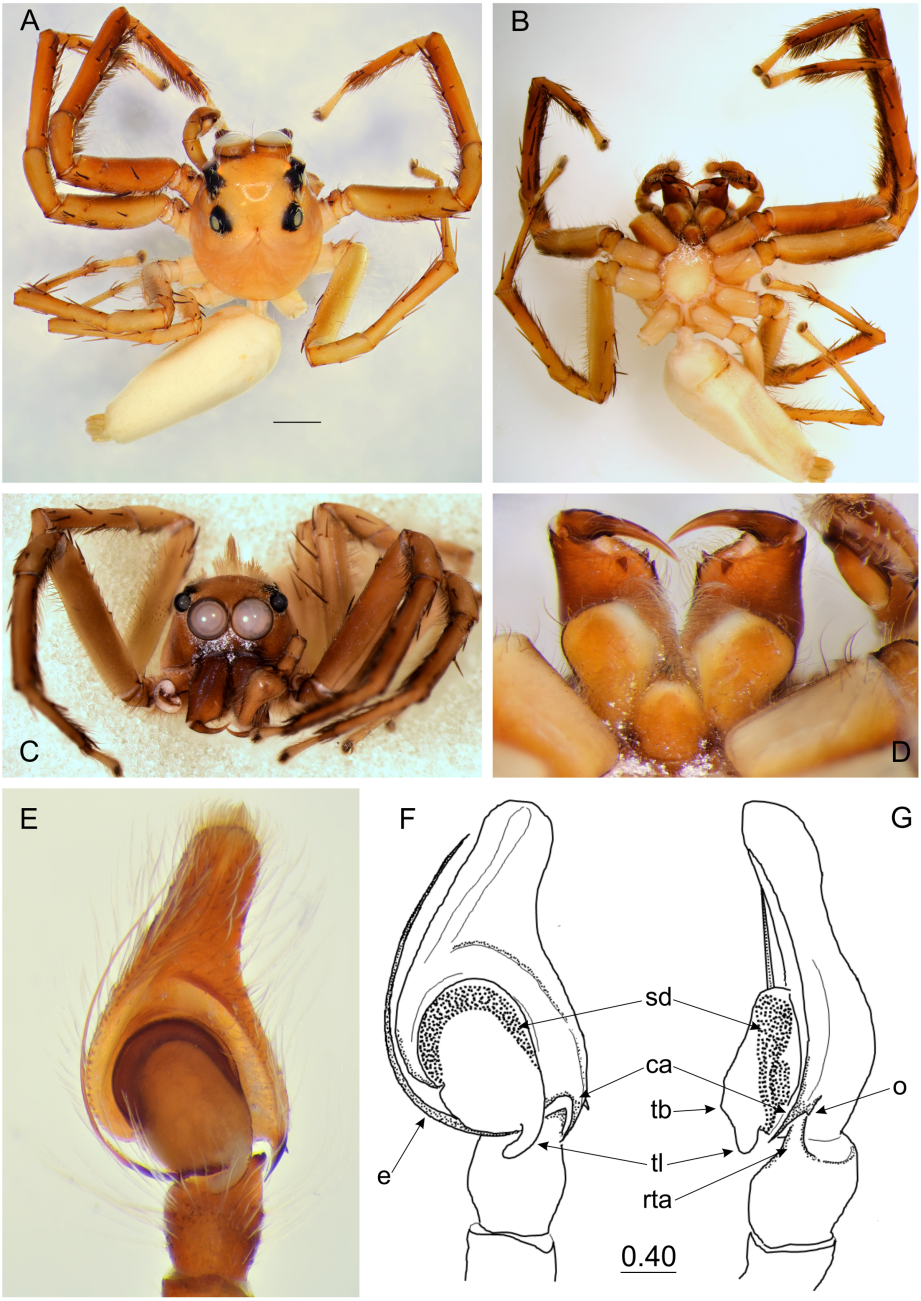
*Epeus daiqini* sp. nov. (male holotype). (A) Dorsal view; (B) ventral view; (C) frontal view; (D) chelicerae, endites and labium; (E–F) right palp ventrally; (G) same, retrolaterally. (Abbreviations: ca, cymbial apophysis; e, embolus; o, outgrowth; rta, retrolateral tibial apophysis; sd, seminal duct; tb, tegular bump; tl, tegular lobe). Scale bar: 1 mm, F–G as in fig.

**Figure 4:**
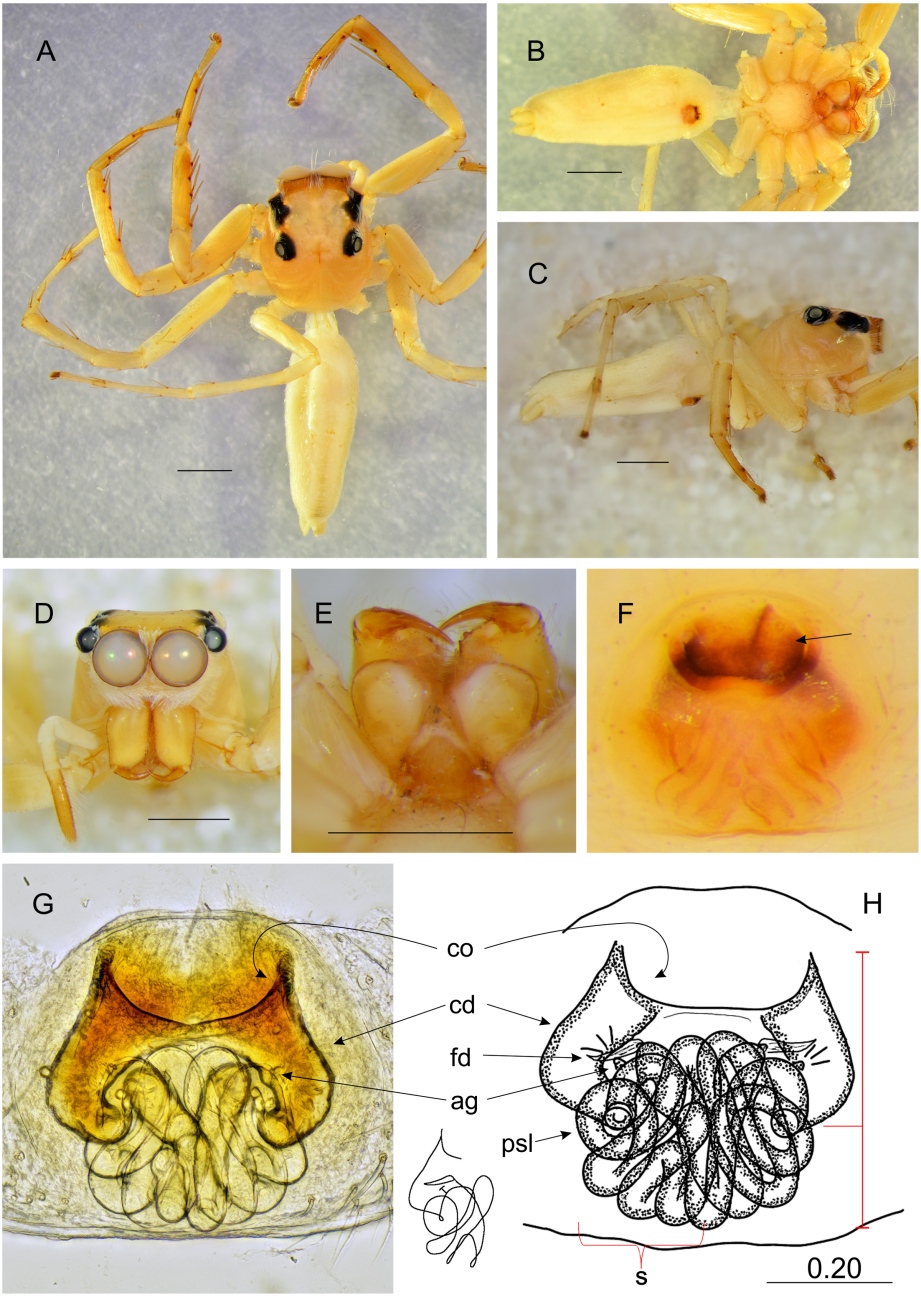
*Epeus daiqini* sp. nov. (female paratype). Salticidae (Arachnida: Araneae) from Thailand (A) dorsal view; (B) ventral view; (C) lateral view; (D) frontal view; (E) chelicerae, endites and labium; (F) epigyne (arrow indicates strongly sclerotized area between copulatory openings); (G–H) vulva. (Abbreviations: ag, accessory gland; cd, copulatory duct; co, copulatory opening; fd, fertilisation duct; psl, proximal spermathecal loop; s, spermatheca). Scale bar: 1 mm, G as in fig.

**Figure 5:**
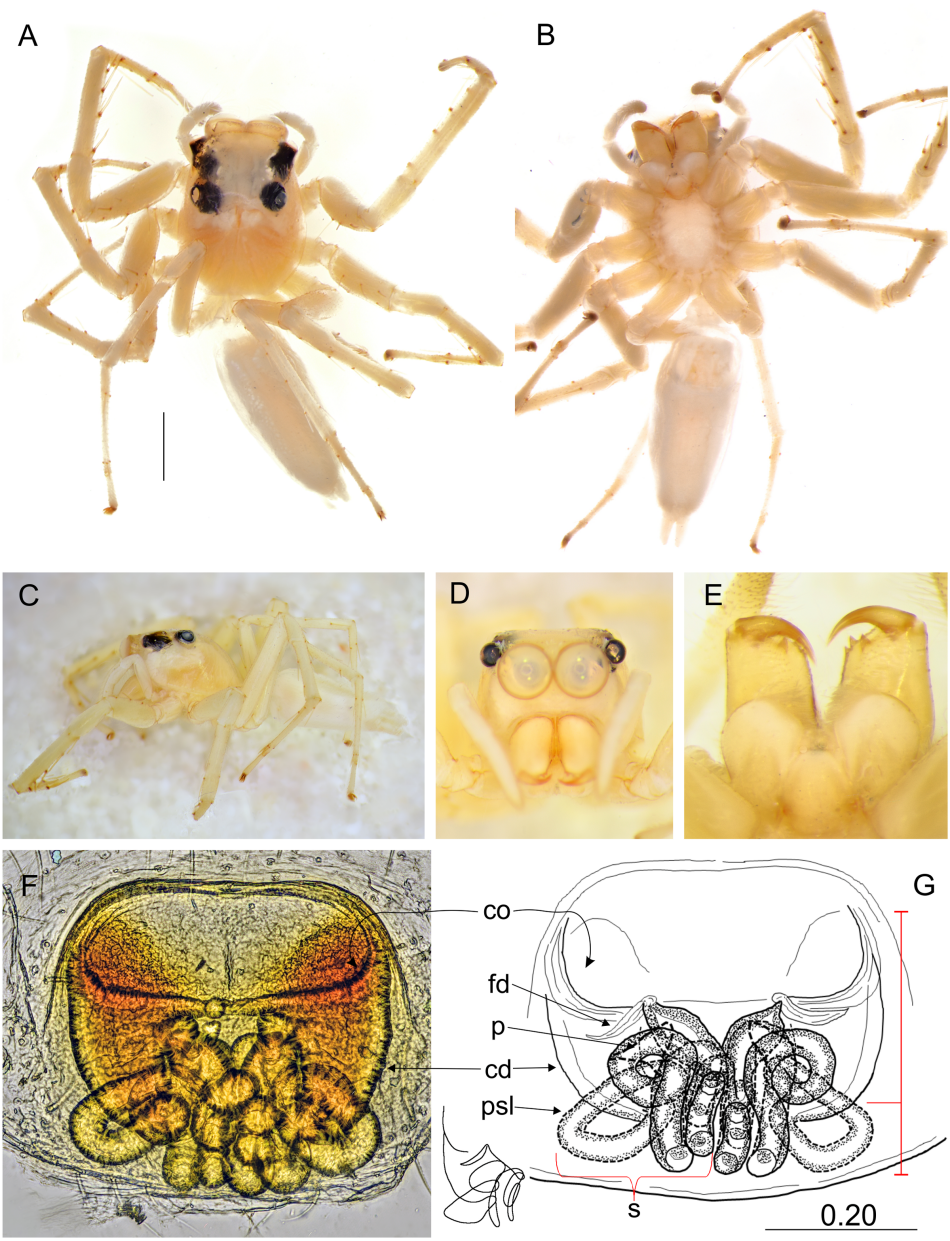
*Epeus pallidus* sp. nov. (female holotype). Salticidae (Arachnida: Araneae) from Thailand. (A) dorsal view; (B) ventral view; (C) lateral view; (D) frontal view; (E) chelicerae, endites and labium; (F–G) vulva. (Abbreviations: cd, copulatory duct; co, copulatory opening; fd, fertilisation duct; psl, proximal spermathecal loop; s, spermatheca). Scale bar: 1 mm, G as in fig.

**Figure 6:**
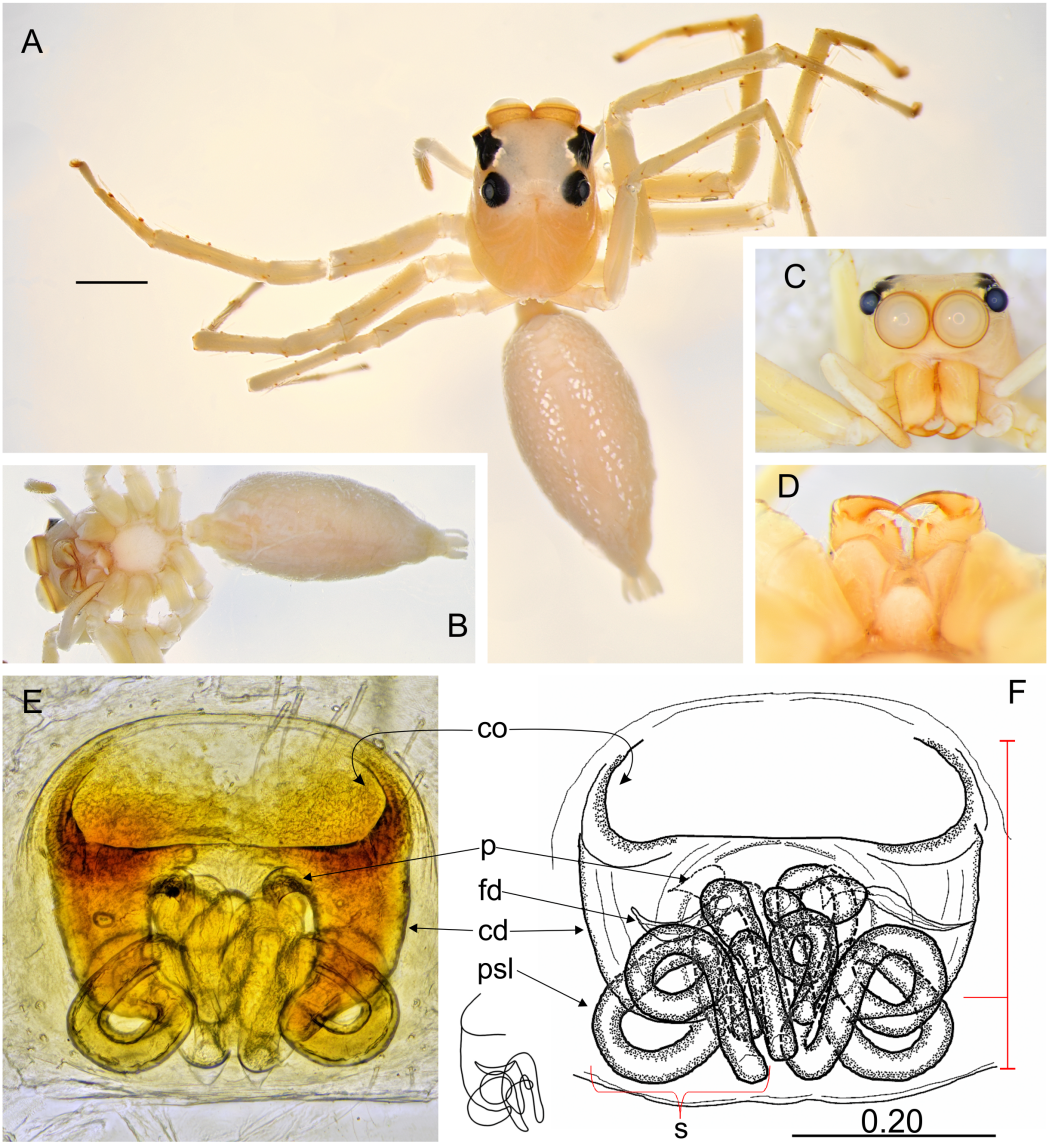
*Epeus szirakii* sp. nov. (female holotype). Salticidae (Arachnida: Araneae) from Thailand. (A) Dorsal view; (B) ventral view; (C) frontal view; (D) chelicerae, endites and labium; (E–F) vulva. (Abbreviations: cd, copulatory duct; co, copulatory opening; fd, fertilisation duct; p, pocket; psl, proximal spermathecal loop; s, spermatheca). Scale bar: 1 mm, F as in fig.

**Figure 7:**
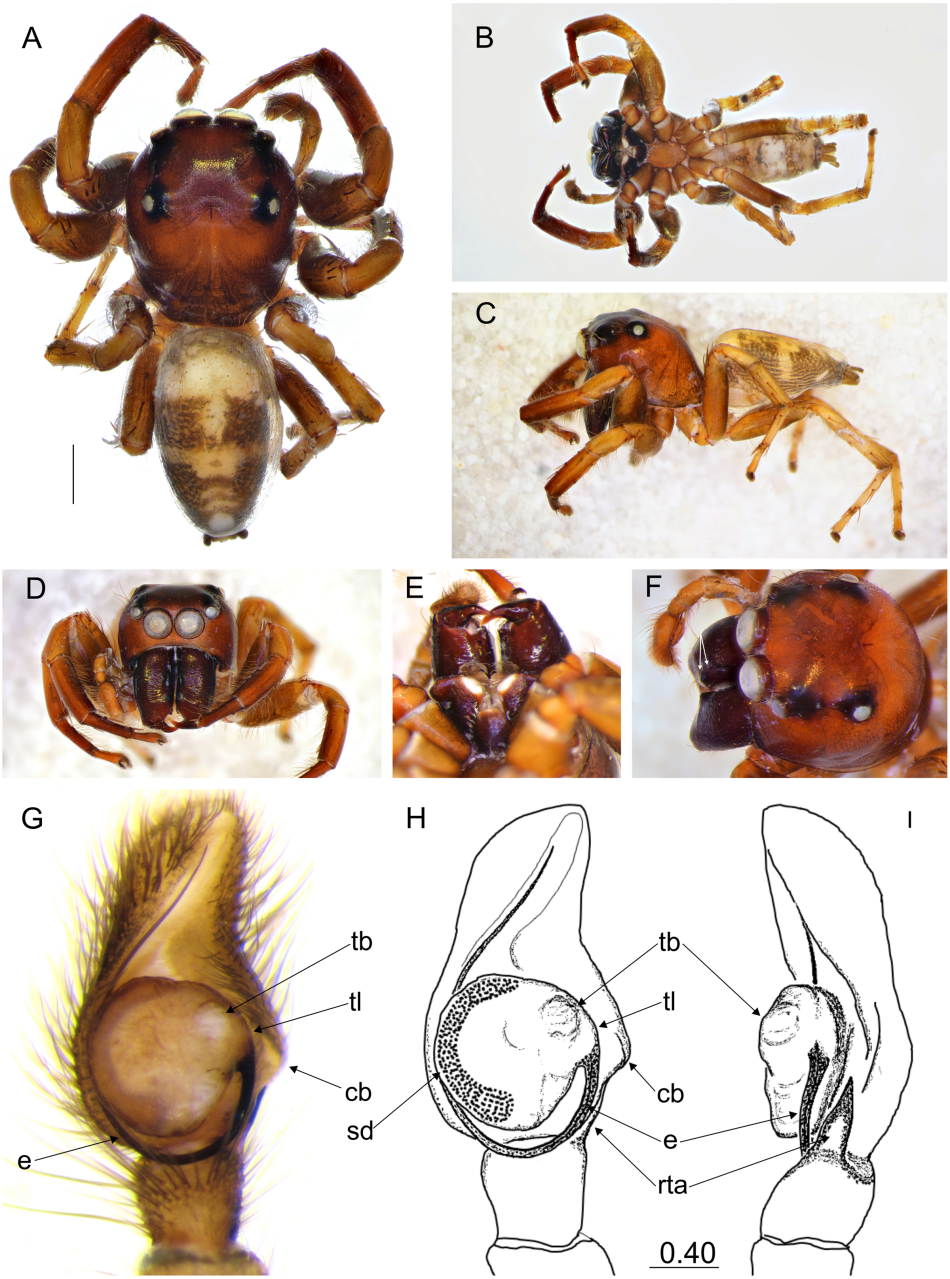
*Ptocasius metzneri* sp. nov. (male holotype). Salticidae (Arachnida: Araneae) from Thailand. (A) Dorsal view; (B) ventral view; (C) lateral view; (D) frontal view; (E) endites and labium; (F) dorsal view of cephalothorax (arrows indicate protuberances on chelicerae); (E–H) right palp ventrally; (I) same, retrolaterally. (Abbreviations: cb, cymbial bump; e, embolus; rta, retrolateral tibial apophysis; sd, seminal duct; tb, tegular bump; tl, tegular lobe). Scale bar: 1 mm, H–I as in fig.

**Figure 8:**
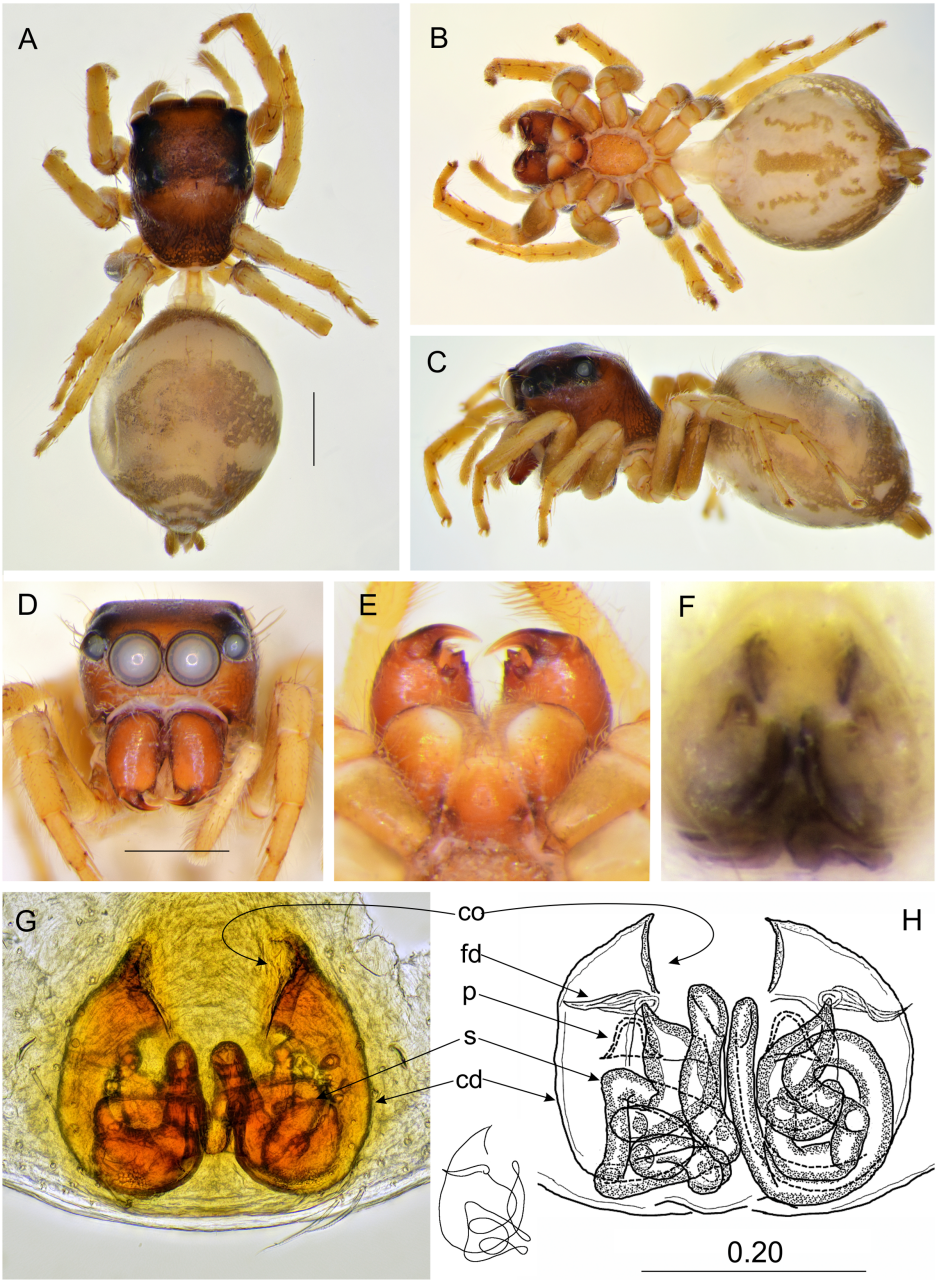
*Ptocasius metzneri* sp. nov. (female paratype). (A) Dorsal view; (B) ventral view; (C) lateral view; (D) frontal view; (E) chelicerae, endites and labium; (F) epigyne; (G–H) vulva. (Abbreviations: cd, copulatory duct; co, copulatory opening; fd, fertilisation duct; p, pocket; s, spermatheca). Scale bar: 1 mm, H as in fig.

**Figure 9:**
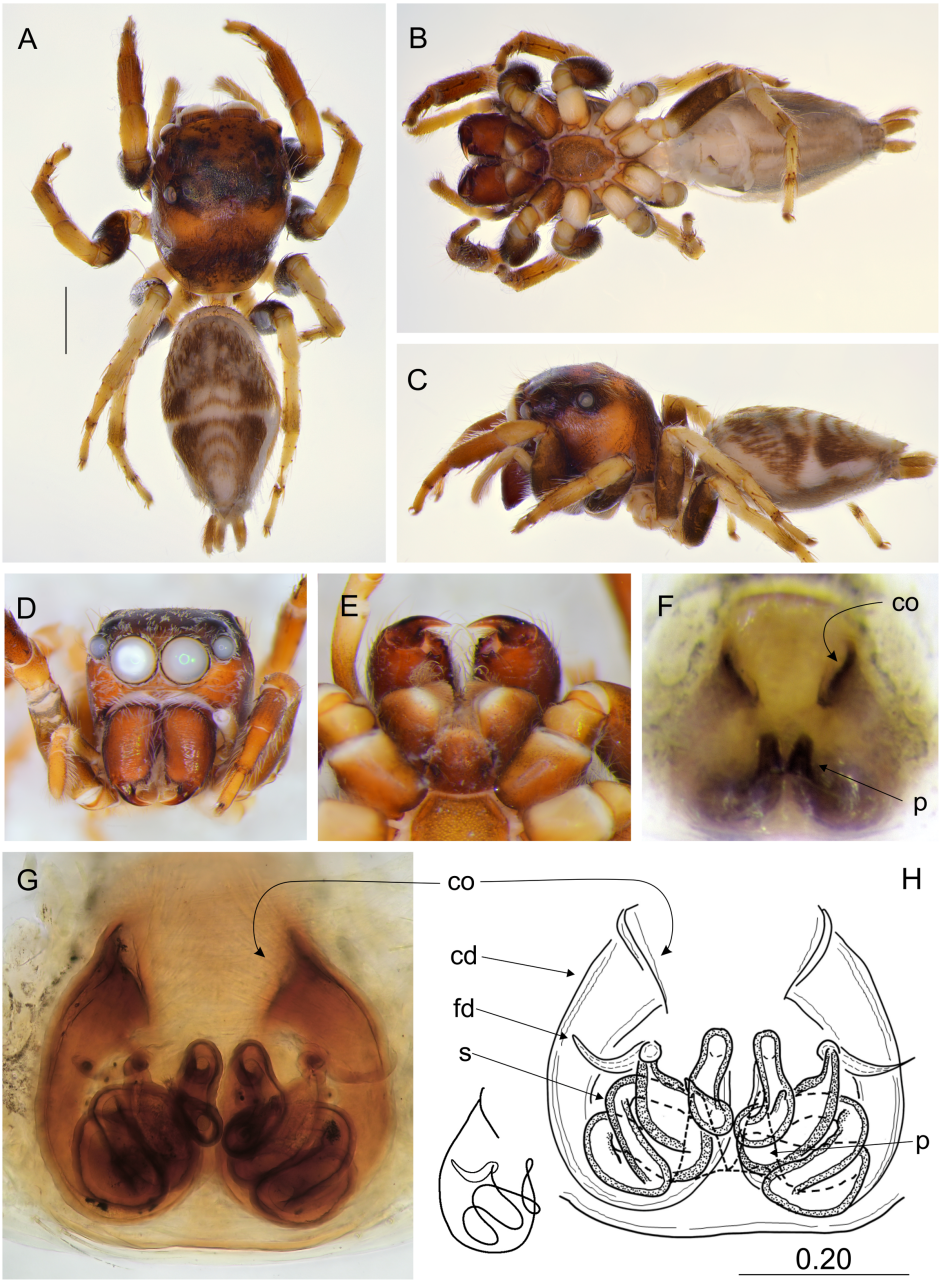
*Ptocasius sakaerat* sp. nov. (female holotype). (A) Dorsal view; (B) ventral view; (C) lateral view; (D) frontal view; (E) chelicerae, endites and labium; (F) epigyne; (G–H) vulva. (Abbreviations: cd, copulatory duct; co, copulatory opening; fd, fertilisation duct; p, pocket; s, spermatheca). Scale bar: 1 mm, H as in fig.

**Figure 10:**
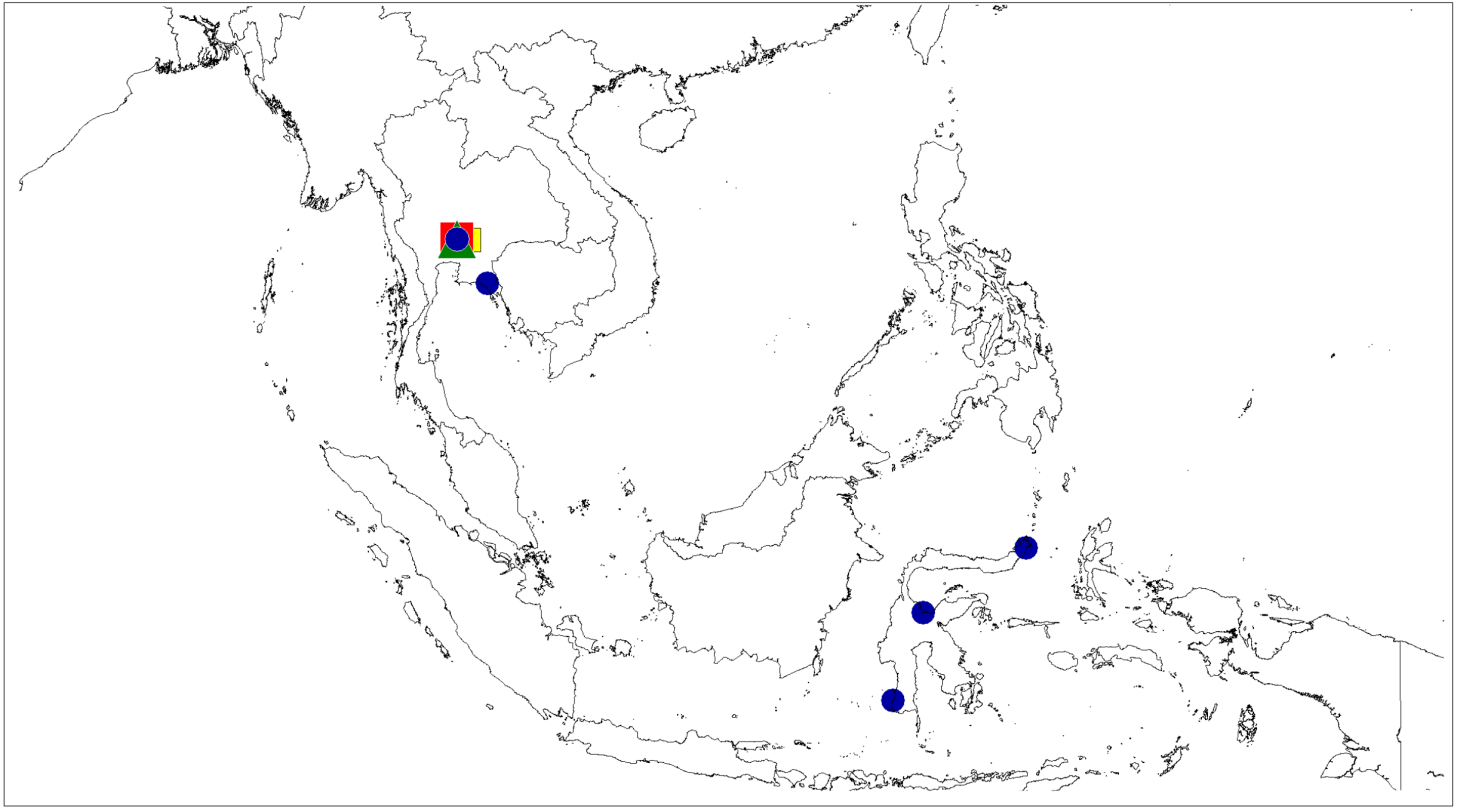
Distributional records of *Epeus*. *E*. *tener* (blue circle), *E*. *daiqini* sp. nov. (yellow square), *E*. *pallidus* sp. nov. (red square), *E*. *szirakii* sp. nov. (green triangle).

**Figure 11:**
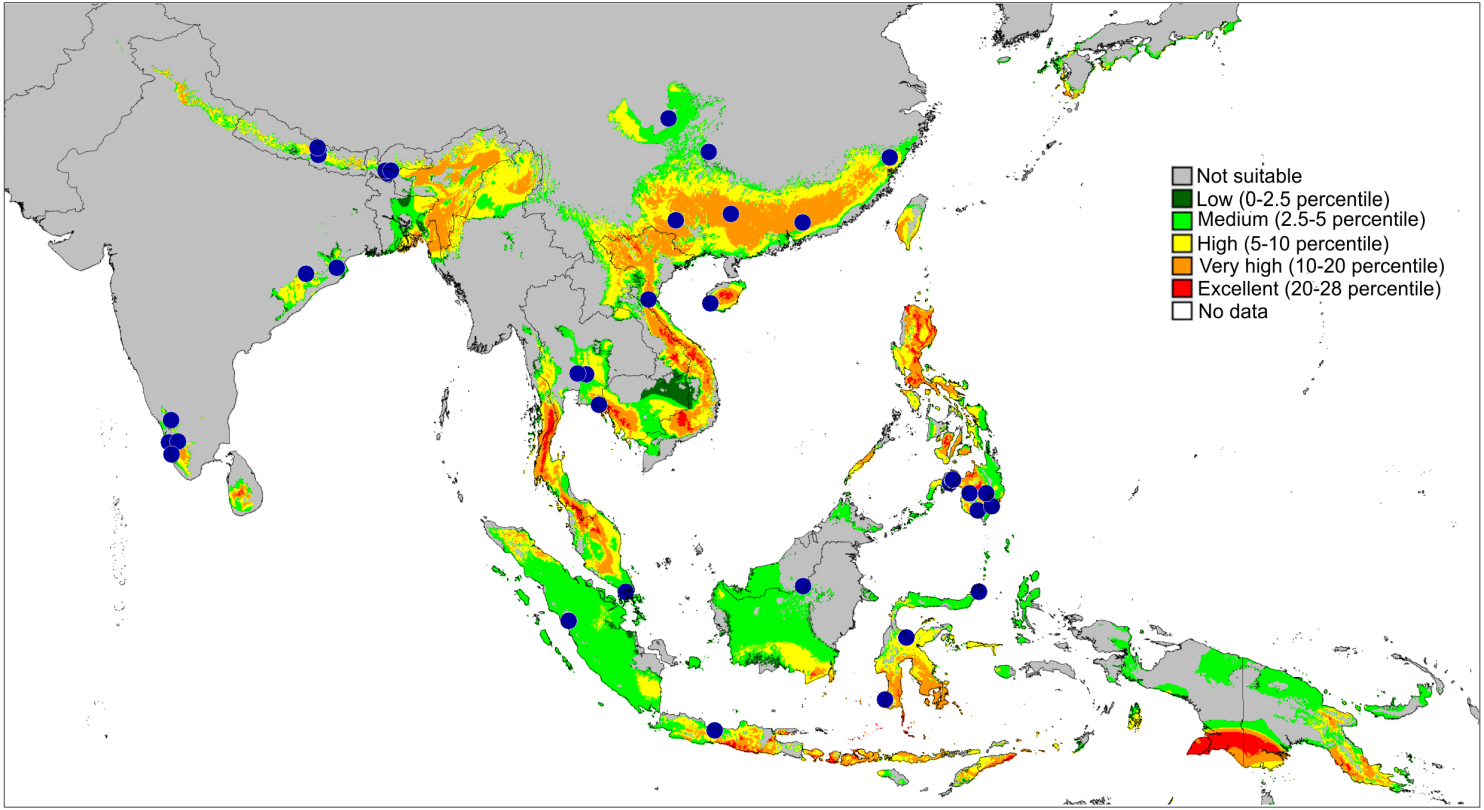
Actual (dots) and predicted distribution of *Epeus* (51 field records). The colours indicate the likelihood of occurrence.

**Figure 12:**
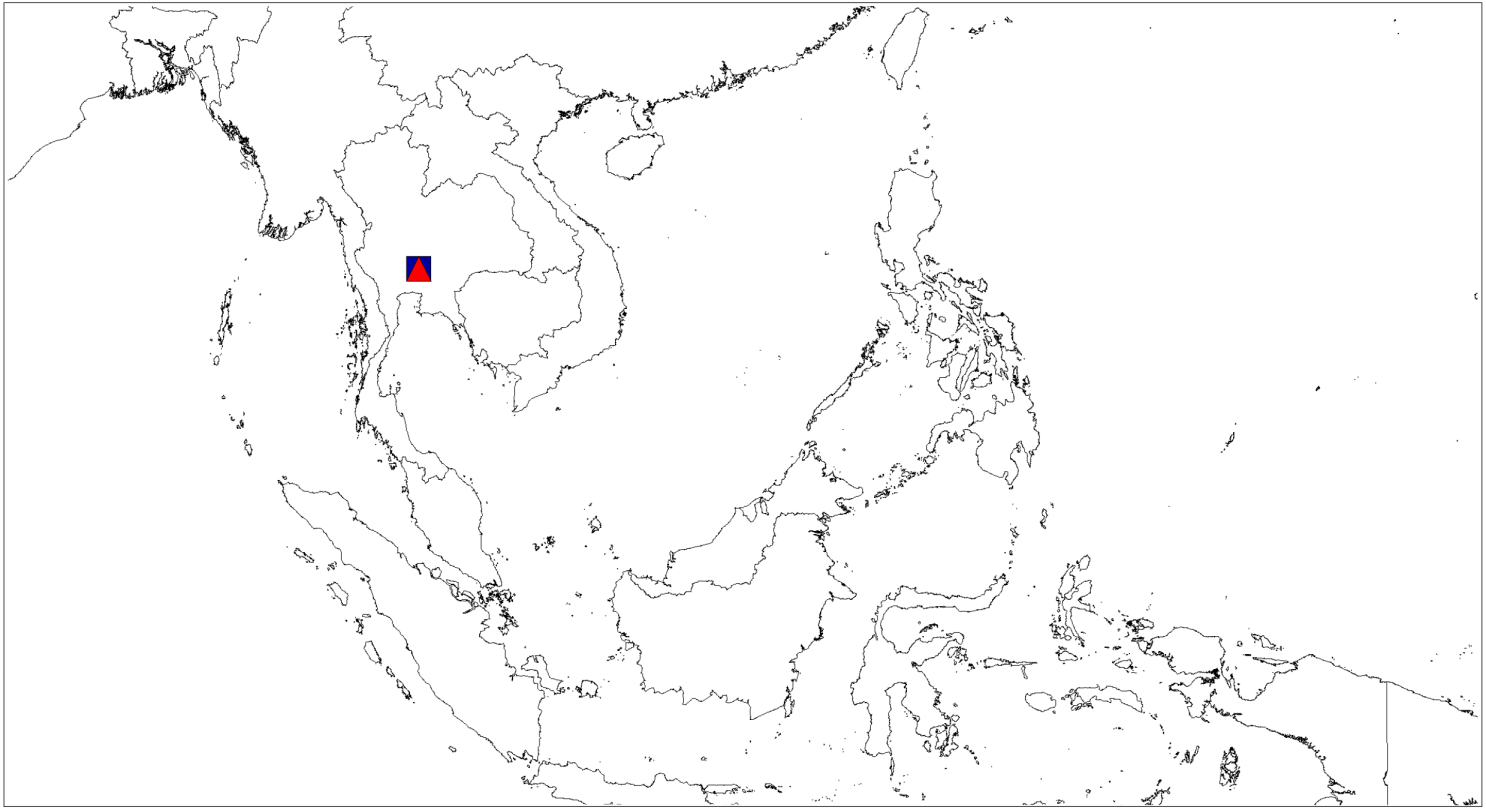
Distributional records of *Ptocasius*. *P*. *metzneri* sp. nov. (blue squre), *P*. *sakaerat* sp. nov. (red triangle).

**Figure 13:**
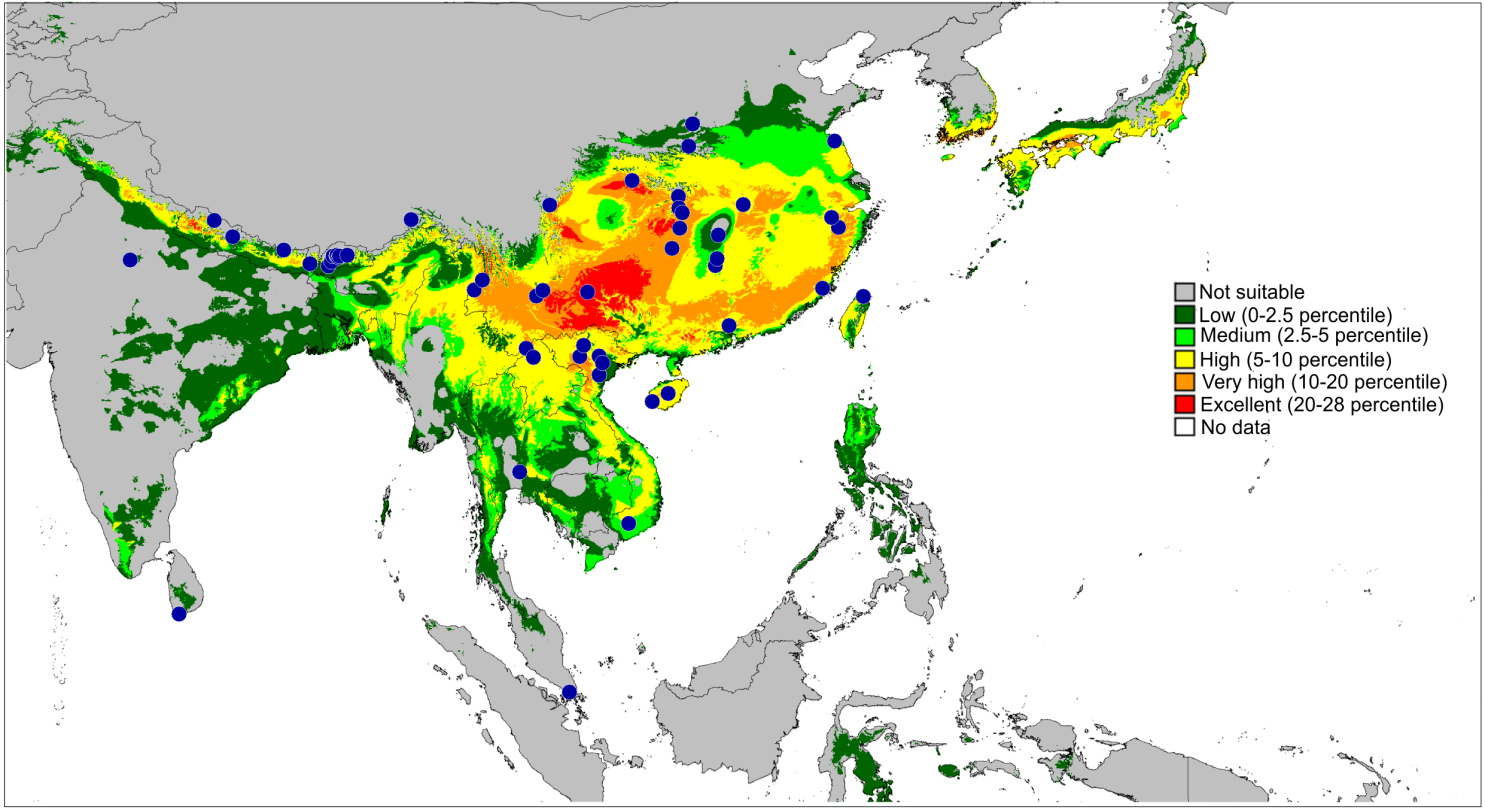
Actual (dots) and predicted distribution of *Ptocasius* (72 field records). The colours indicate the likelihood of occurrence.

The specimens included in this study were collected by Dr. Christa Deeleman-Reinhold (CHD) and E. Horváth and Gy. Sziráki. The type material has been deposited in the Naturalis Biodiversity Centre in Leiden, The Nederlands (RNMH) and the Hungarian Natural History Museum, Budapest, Hungary (HNHM). The examination methods follow the protocol proposed by Żabka (1991) and diagnostic characters are illustrated here in Figs. 1–9. Leg spination follows the system used by Ono (1988). The embolic base and tegular apophyses have been described using clock position. The drawings were made using a grid system. The photographs were taken with Nikon D5200 camera and Nikon SMZ1000 stereomicroscope, and were digitally processed with ZOOMBROWSER and HELICON FOCUS software. The actual and predicted distributional maps were generated with DIVA-GIS 7.5 bio-climatic software (Hijmans, Guarino & Mathur, 2012); our model has been generated with 51 field records for the genus *Epeus* and 72 for *Ptocasius* (Figs. 10–13) and met the requirements for the software (Hernandez et al., 2006). The following environmental variables were used in the analysis: annual mean temperature, mean monthly temperature range, isothermality, temperature seasonality, maximum temperature of warmest month, minimum temperature of coldest month, temperature annual range, mean temperature of wettest quarter, mean temperature of driest quarter, mean temperature of warmest quarter, mean temperature of coldest quarter, annual precipitation, precipitation of wettest month, precipitation of driest month, precipitation seasonality, precipitation of wettest quarter, precipitation of driest quarter, precipitation of warmest quarter, precipitation of coldest quarter. Only selected recent papers have been cited treating these two taxa, for complete bibliography and documentation of particular species see Prószyński (2019), Metzner (2020) and WSC (2020). The electronic version of this article in Portable Document Format (PDF) will represent a published work according to the International Commission on Zoological Nomenclature (ICZN), and hence the new names contained in the electronic version are effectively published under that Code from the electronic edition alone. This published work and the nomenclatural acts it contains have been registered in ZooBank, the online registration system for the ICZN. The ZooBank LSIDs (Life Science Identifiers) can be resolved and the associated information viewed through any standard web browser by appending the LSID to the prefix http://zoobank.org/. The LSID for this publication is: urn:lsid:zoobank.org:pub:0B55634A-1D0D-49BD-BE23-669C3630715C. The onlineversion of this work is archived and available from the following digital repositories: PeerJ, PubMed Central and CLOCKSS.

## Results & Discussion

**Table:** 

Family Salticidae Blackwall, 1841
Genus *Epeus* Peckham & Peckham, 1886
*Evenus*Simon, 1877: 58 (preoccupied)
*Viciria*Thorell, 1887: 393 [part]; (Simon, 1903): 751 [part].
*Epeus*Peckham & Peckham, 1886: 334; Prószyński, 1984: 403, 1992: 171; Żabka, 1985: 214; Peng et al., 1993: 47; Barrion & Litsinger, 1995: 67; Peng & Li, 2002: 385; Zhang, Song & Li, 2003: 192; Jastrzębski, 2010: 115; Prószyński & Deeleman-Reinhold, 2012: 36; Meng, Zhang & Shi, 2015: 147; Malamel et al. 2019: 269; Prószyński, 2019; WSC, 2020.

**Type species**: *Evenus tener* (Simon, 1877), from Basilan Island (Philippines), by monotypy.

**Diagnosis**. In comparison with related *Telamonia* (Thorell, 1887), males with crest of carapace setae arranged in mohawk manner, and cymbium with an apophysis instead of strong bristles. Unlike *Plexippoides,* the body is very elongate and the cymbium much narrower. Female genitalic structures in the three mentioned genera rather similar and of limited diagnostic value. In the genus *Epeus*, the epigynal plate with large atrium, membranous copulatory ducts running posteriorly and multi-looped spermathecae. Accessory glands present, but not always clearly visible.

**Description.** Spiders 4–8 mm long, alive individuals lush green, yellow to orange (Fig. 1). Carapace height about 80% of length, in some species eye field in males with fringe/crest of hairs upright in a mohawk style (Figs. 1B and 3C). Thoracic slope distinctive. AME much larger than ALE, the latter slightly behind AME. Abdomen elongate, pale or green with translucent guanine patches. Spinnerets not distinctive. Metatarsi, tibiae and patellae of first and second leg in males usually with fringes of hairs (Fig. 3B). Chelicerae unidentate. Endites in some males with a small anterolateral protuberance (pr, Fig. 2E). Palpal cymbium with uni- or bicuspidate apophysis (ca), their edges serrated (Fig. 2F) or smooth (Figs. 3E–3F). Tegulum with a posterior lobe (tl, Figs. 2F–2G and 3G) and bump (tb, Figs. 2G and 3G). Embolus (e) filamentous and long, set at the posterior part of tegulum (Figs. 2F, 3E–3F). Retrolateral tibial apophysis single (rta, Figs. 2G and 3G). Females usually lighter in coloration. Epigynes (Figs. 4F–4H, 5F and 6E) with a single anterior atrium, in some species with pockets (p, Figs. 5F–5G and 6E–6F). Copulatory openings (co) creviced, oriented anteriorly (e.g., *E*. *pallidus* sp. nov., *E*. *szirakii* sp. nov., *E*. *sumatranus* Prószyński & Deeleman-Reinhold, 2012) or towards each other (e.g., *E*. *alboguttatus* (Thorell, 1887), *E*. *indicus* Prószyński, 1992), with intermediate stages (e.g., *E*. *daiqini* sp. nov., *E*. *tener*). Copulatory ducts (cd) wide and membranous, joining multi-coiled spermathecae (s); the boundary between both structures indistinct; with more or less visible loop-like duct (psl).

**Relationships.** According to Prószyński & Deeleman-Reinhold (2012) the genus is closely related to *Plexippoides* (Prószyński, 1984), sharing morphological similarities in male genitalia, especially in the form of the cymbial apophysis, tegular lobe and embolus. On the basis of molecular quantitative analyses, Maddison (2015) proposed close relationships between *Epeus* and *Plexippoides*, *Ptocasius*, *Telamonia* and *Yaginumaella*. We basically agree with both hypotheses and, on the basis of genitalic structure, we consider *Epeus*, *Plexippoides* and *Telamonia* to be the close relatives.

**Distribution and biology.** The genus *Epeus* is a vegetation dweller, distributed from subtropical Himalayan valleys, through India, Indochina, southern China, Philippines and Sunda Archipelago (Fig. 11). The map of the predicted distribution (Fig. 11) suggests the presence of the genus in other areas, including New Guinea, however, despite the review of major collections from New Guinea (MŻ) no evidence was found to support such a prediction.

### *Epeus tener* (Simon, 1877)

**Table:** 

(Figs. 2 and 10)
*Evenus tener*Simon, 1877: 59.
*Epeus tener*Peckham & Peckham, 1886: 334.
*Viciria cristata*Thorell, 1887: 393.
*Viciria tenera*Simon, 1903: 743, 752; Merian, 1911: 315.
*Epeus tener*Prószyński, 1984: 409; Żabka, 1985: 216; Prószyński & Deeleman-Reinhold, 2012: 38, Prószyński, 2017: 33.

**Diagnosis**. *Epeus tener* can be distinguished from all other species by having an embolus serrated at the base and a tegular apophysis base fixed at 4.30 o’clock, with an additional outgrowth set at 7 o’clock. Cymbial apophysis pointed, its edges serrated. Epigynal plate widening posteriorly. Internal genitalia similar in structure to *E*. *pallidus*, but copulatory openings strongly sclerotized, oriented more anteriorly, proximal spermathecal loop different in course.

**Material:** 1♂, Prov. Prachin Buri, Sakaerat Environmental Research Station, Thailand, [14°30′0″N, 100°52′0.12″E], No 26, 2 June 2001, leg. E. Horváth & Gy. Sziráki, HNHM Araneae-9630.

**Remark**. The species has already been recorded in Thailand (as *Viciria cristata*, (Thorell, 1887). Here we give new locality and complete the description with habitus and palp images, drawing and a distribution map.

**Distribution**. Thailand, Vietnam, and Indonesia (Java, Sulawesi) (Fig. 10).

### *Epeus daiqini* sp. nov.

(Figs. 3–4, 10)

**Material:** Holotype ♂, [Prov. Nakhon Nayok], Khao Yai National Park, Thailand, [14°26′29″N, 101°22′11″E], sec. forest, 1050 m, 2 March 1986, coll. and leg. C. Deeleman-Reinhold, RNMH.ARA.18409, Paratypes: 3 ♀ ♀, 3 juv., same data, RNMH.ARA.18410.

**Etymology.** The specific epithet is a patronym in honour of Prof. Daiqin Li (National University of Singapore) in recognition of his research on the behaviour of Salticidae.

**Diagnosis**. Males differ from similar/related species *E*. *tener*, *E*. *furcatus* (Zhang, Song & Li, 2003), and *E*. *bicuspidatus* (Song, Gu and Chen, 1988) as missing embolic serration, and the presence of a small outgrowth on the retrolateral cymbial apophysis. Female copulatory openings more widely separated from each other than in *E*. *alboguttatus*, but less distant than in *E*. *pallidus* and *E*. *szirakii*. Females differ from *E*. *tener* by the orientation of copulatory openings (more diagonal in *E*. *daiqini*). Copulatory ducts do not reach epigastric fold, and proximal spermathecal loop (psl) less distinctive than in other species, accessory glands clearly visible.

**Description.** Male holotype (Figs. 3A–3G). Carapace orange with cephalic crest (Fig. 3C), covered with scale-like light hairs. Surroundings of AME brown, other eye surroundings black. Abdomen yellowish, spinnerets orange. Clypeus and chelicerae dark brown. Endites and labium orange, with paler tips, the former with no protuberance. Sternum yellow, with darker margin. Ventral abdomen yellowish. Legs light brown, the first pair darker and stronger than the others, with a fringe of brown setae on ventral patella, tibia and metatarsus. Spines numerous on all legs. Spination of leg I: tibia ventral 2–2–2–2, retrolateral 1–1–0–1, metatarsus ventral 2–2, pro- and retrolateral 1–1. Cymbium apically slightly curved, its apophysis with a short outgrowth (Fig. 3G). Tegulum with a small bump and with a distinctive, posterior finger-like tegular lobe. Retrolateral tibial apophysis single (Fig. 3G). Dimensions: CL 3.40, CW 2.80, CH 2.00, EFL 1.40, AEW 2.10, PEW 1.90, AL 4.40, AW 1.70.

Female paratype (Figs. 4A–4H). Carapace yellowish, eye field paler, with translucent guanine deposits. Abdomen yellowish, slightly darker along median part, with lateral guanine deposits. Clypeus yellow, densely covered with whitish scale-like hairs. Chelicerae, endites and labium yellow. Sternum and ventral abdomen whitish. Spinnerets pale yellowish. Legs yellow, long and spiny, palps yellowish with darker tarsi. Spination of leg I: tibia ventral 2–2–2–2, metatarsus ventral 2–2. Epigynal plate rather round, atrium ovoid (Figs. 4F–4H), copulatory openings with joined posterior margins, oriented antero-diagonally. The area between openings strongly sclerotized (Fig. 4F). Pockets absent. Copulatory ducts relatively short and do not reach the epigastric fold area. Accessory glands visible. Dimensions: CL 2.50 −2.60, CW 1.90 −2.10, CH 1.50 −1.70, EFL 1.20 −1.25, AEW 1.80 −1.85, PEW 1.55 −1.60, AL 3.20 −3.60, AW 1.40 −1.45.

**Distribution.** Known only from the type locality in Thailand (Fig. 10).

### *Epeus pallidus* sp. nov.

(Figs. 5 and 10)

**Material:** Holotype ♀, Prov. Prachin Buri, Sakaerat Environmental Research Station, Thailand, [14°30′0″N, 100°52′0.12″E], No 76, evergreen forest, 01–16.06.2001, leg. E. Horváth & Gy. Sziráki, HNHM Araneae-9631. Paratype: 1 ♀ No 50 (same data), HNHM Araneae-9632.

**Etymology.** The specific epithet, “pallidus”, refers to the spider’s pale coloration.

**Diagnosis.** Can be distinguished from all other species by rather rectangular epigynal plate, and large and wide atrium. Copulatory openings widely spaced, more distant than in *E*. *albus* (Prószyński, 1992), but similar to *E*. *szirakii*. In comparison with the latter species proximal spermathecal loops (psl) different in shape.

**Description.** Female holotype (Fig. 5). Carapace yellowish, eye field paler, with visible guanine deposits. ALE, PME and PLE surrounded with black. Clypeus whitish, below AME covered with white scale-like hairs. Chelicerae, endites and labium, sternum and pedipalps very pale. Abdomen, ventral abdomen and spinnerets almost white. Legs yellowish. Spination of leg I: tibia ventral 2–2–2–2, metatarsus ventral 2–2. Epigynal plate intermediate, between ovoid and rectangular. Atrium wide, copulatory openings far apart from each other, crescent-like. Epigynal pockets poorly visible, similar to *E*. *szirakii*, located centrally, their openings oriented postero-laterally (Figs. 5F–5G). Copulatory ducts similar to *E*. *szirakii.* Proximal spermathecal loops distinctive as in *E*. *szirakii*, but of different shape. Accessory glands not visible. Dimensions: CL 2.70, CW 2.10, CH 1.25, EFL 1.25, AEW 1.80, PEW 1.60, AL 3.30, AW 1.40.

Male unknown.

**Distribution**. Known only from type locality in South Thailand (Fig. 10).

### *Epeus szirakii* sp. nov.

(Figs. 6 and 10)

**Material:** Holotype 1 ♀, Prov. Prachin Buri, Sakaerat Environmental Research Station, Thailand, [14°30′0″N, 100°52′0.12″E], No 65, evergreen forest, 01–16.06.2001, leg. E. Horváth & Gy. Sziráki, HNHM Araneae-9633.

**Etymology.** The specific epithet is patronym in honour of György Sziráki (Hungarian Natural History Museum, Budapest, Hungary), the Hungarian entomologist and collector of the material studied here.

**Diagnosis.** In comparison with other similar species (*E*. *pallidus*, *E*. *mirus* (Peckham & Peckham, 1907), *E*. *sumatranus*) it differs in lengths and shape of internal genitalia (Figs. 6E–6F). Copulatory openings widely separated as in *E*. *pallidus* sp. nov., also epigynal pocket similar, but proximal spermathecal loops of different shape.

**Description.** Female holotype (Figs. 6A–6F). Carapace yellowish, eye field almost white, with lateral guanine deposits, surroundings of eyes black, with yellow around AME. Abdomen whitish, with numerous bright guanine deposits. Clypeus whitish, centrally covered with white hairs. Chelicerae, endites and labium grayish-yellow. Sternum whitish. Ventral abdomen pale grayish. Spinnerets white. Legs yellow-white, spiny. Spination of leg I: tibia ventral 2–2–2–2, metatarsus ventral 2–2. Palps whitish, with yellowish tarsi. Epigyne with large and wide atrium, with two small central pockets. Copulatory openings widely separated, crescent-like. Copulatory ducts as in other species, proximal spermathecal loops very distinctive (Figs. 6E–6F). Dimensions: CL 2.50, CW 1.95, CH 1.50, EFL 1.20, AEW 1.70, PEW 1.50, AL 3.75, AW 1.90.

Male unknown.

**Distribution**. Known only from the type locality in South Thailand (Fig. 10).

### Genus *Ptocasius* (Simon, 1885)

*Ptocasius* Simon, 1885: 34; Żabka, 1985: 440; Peng et al., 1993: 196; Zhu & Zhang, 2011: 509; Prószyński, 2019, WSC, 2020.

*Yaginumaella* [part]: Prószyński, 1979: 319–320; Żabka, 1980: 371, 1981: 6–7.

**Type species**: *Ptocasius weyersi* (Simon, 1885), from Fort de Kock (Sumatra), by monotypy.

**Diagnosis**. Unlike related *Epeus, Plexippoides* and *Telamonia* the body is not elongate, and abdominal pattern with chevrons or transverse dark and light stripes. Cymbium rather wide, neither with apophysis nor with strong bristles. In some species a cymbial bump present. Tegulum either round or elongate. Tegulum shape, embolus and tibial apophysis similar to related *Epeus*, *Telamonia* and *Plexippoides*.

**Description.** The body not distinctive, 5–7 mm long. The colour pattern usually with abdominal chevrons or contrasting, transverse stripes and patches. Cephalothorax moderately high, longer than wide. Eyes in three rows. Thoracic slope distinctive. Fovea between or behind PLE. Chelicerae unidentate (Figs. 7E and 8E). Abdomen elongate ovoid. Legs not distinctive, the first stronger than the others. Tegulum of palpal organ ovoid or elongate, with a lobe (tl) located either posteriorly or at the base of embolus (Figs. 7G–7H), but never as distinctive as in *Epeus*. Additionally, in some species tegular bump present. Spermophore not meandering. Embolus varies in length and thickness, being the shortest in *P*. *thakkholaica* (Żabka, 1980) comb. nov. and the longest in *P*. *weyersi*, *P*. *paraweyersi* Cao & Li, 2016 and *P*. *stemmleri* (Żabka, 1981) comb. nov. Cymbium much wider than tegulum, without apophysis. Tibial apophysis (rta) single (Fig. 7I). Epigyne usually with a pair of pockets located in different positions: from extreme anterior as in *P*. *pseudoflexa* (Liu, Yang & Peng, 2016) comb. nov.*,* to posterior as in *P*. *hybrid* (Żabka, 1981) comb. nov. In *P*. *cambridgei* (Żabka, 1981) comb. nov., *P*. *thakkholaica* (Żabka, 1980) comb. nov. and *P*. *wangdica* (Żabka, 1981) comb. nov., the pockets are distant from one another, while in *P*. *falcate* (Zhu et al., 2005) comb. nov. and *P*. *pulchella* (Li et al., 2018) comb. nov. they are nearly merging. Copulatory openings creviced, located anteriorly and oriented anteriorly, diagonally or towards each other. Copulatory ducts wide and membranous. Spermathecae multi-coiled.

**Relationships**. On the basis of a molecular analysis, Maddison (2015) placed *Ptocasius* in the Plexippini tribe, together with *Epeus, Plexippoides*, *Telamonia*, *Yaginumaella* and 42 other genera. Prószyński (2017), using morphological characters, agrees that there is a close relationship between *Ptocasius* and *Yaginumaella*. We support both views, and, after the comparison of our material with *Ptocasius weyersi*—the type species of the genus, we have transferred 37 species of *Yaginumaella* (most of them described by MŻ) to the genus *Ptocasius*. Our decision is based on homologies/similarities in genitalic structures and priority of the generic name *Ptocasius*. At this stage we do not discuss the validity of *Yaginumaella* as such. Indeed, the genus presents some differences in genitalia, however its status remains an open issue.

**Distribution and biology.** Newly formulated, the genus *Ptocasius* includes 52 species distributed in two major diversity centres. The richest diversity is located in Nepal–Buthan Himalayas (from 700 to 3,400 m) and comprises 27 species characterised by small morphological gaps in the genitalic structures and found in narrow altitudinal vegetation zones. Prószyński (1980) suggested that such a case is the result of high speciation rate in unique ecological, historical and bioclimatic conditions. Such a phenomenon is widely known in tropical and subtropical mountains for many groups of animals and plants (Mani, 1974; Nice et al., 2013; Ebersbach, Schnitzler & Favre, 2017).

The second major diversity centre includes 23 species found in SE China, Hong Kong, Vietnam, Myanmar, Thailand and Malay Peninsula. A map of the predicted distribution (Fig. 13), generated for all species and all available localities (72 records), suggests that the number for the latter area is seriously underestimated.

There are two hypotheses about possible relationships between these two groups:

1. The Himalayan lineage is derived from tropical (SE Asian) ancestors and speciated in great numbers on the spot. The isolation in various Himalayan valleys and the changing bioclimatic conditions found in different altitudinal zones certainly created ideal conditions for such an intense radiation.
2. The SE Asian group of species is derived from Himalayan ancestors.

The relationships of the genus *Ptocasius* to related genera suggest the first hypotheses to be more likely, but to verify the case it will be necessary to perform molecular analyses to estimate relationships and dates of divergence within the genus.

**Figure 14:**
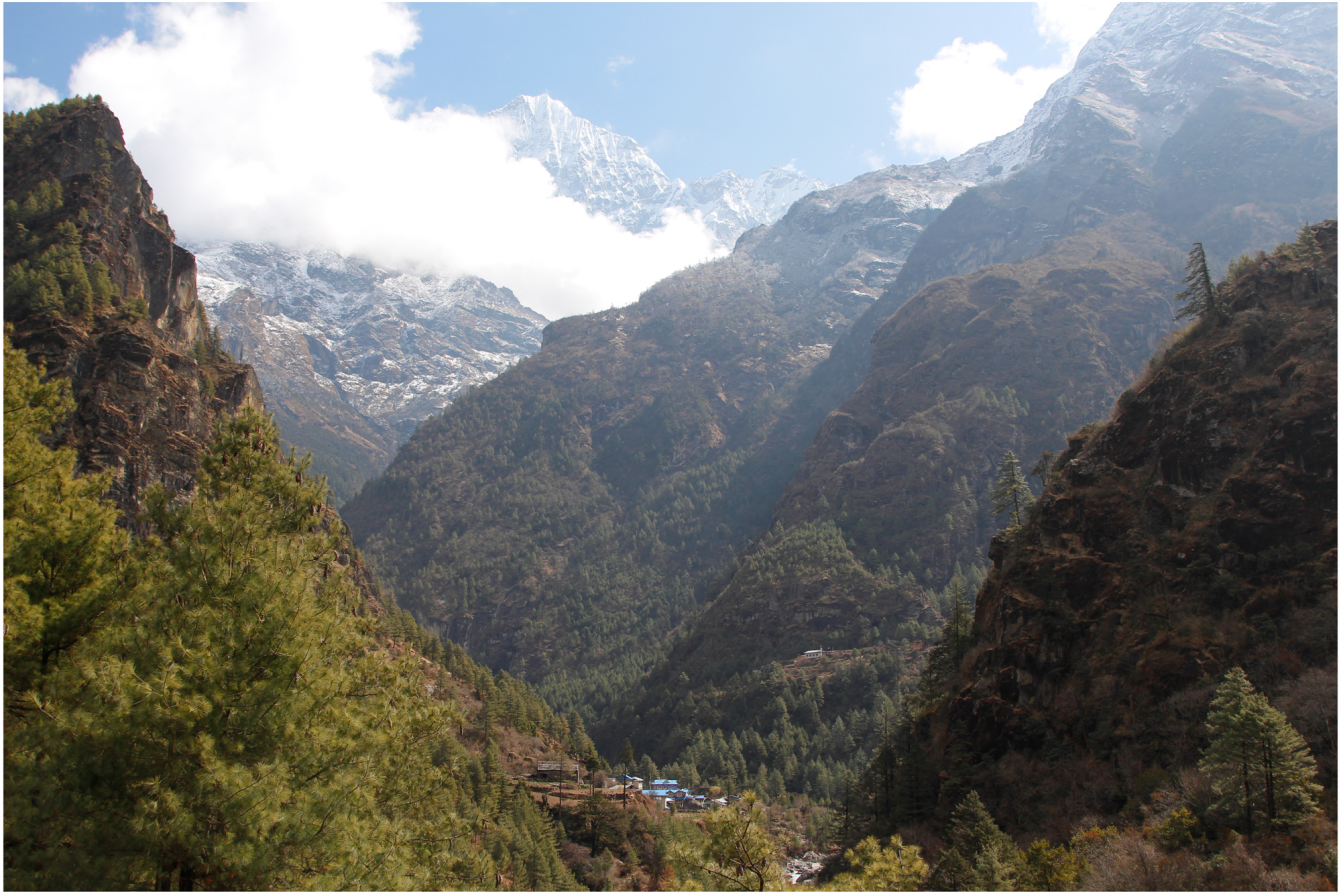
In the Himalayan valleys the vegetation zones depend on altitude and exposure. (Nepal: Phakding image credit: Maciej Bartos).

Species of the genus *Ptocasius* can be found in evergreen forests of *Quercus*, *Rubus*, *Castanopsis*, and different types of *Rhododendron* and bamboo forests at higher altitudes (Fig. 14).

The list of species transferred to *Ptocasius*.

1. *Ptocasius badongensis* (Song & Chai, 1992) comb. nov.
2. *Ptocasius bhutanica* (Żabka, 1981) comb. nov.
3. *Ptocasius bilaguncula* (Xie & Peng, 1995) comb. nov.
4. *Ptocasius bulbosa* (Peng, Tang & Li, 2008) comb. nov.
5. *Ptocasius cambridgei* (Żabka, 1981) comb. nov.
6. *Ptocasius falcate* (Zhu et al., 2005) comb. nov.
7. *Ptocasius gogonaica* (Żabka, 1981) comb. nov.
8. *Ptocasius helvetorum* (Żabka, 1981) comb. nov.
9. *Ptocasius hubeiensis* (Li et al., 2018) comb. nov.
10. *Ptocasius hybrid* (Żabka, 1981) comb. nov.
11. *Ptocasius incognita* (Żabka, 1981) comb. nov.
12. *Ptocasius intermedia* (Żabka, 1981) comb. nov.
13. *Ptocasius lushiensis* (Zhang & Zhu, 2007) comb. nov.
14. *Ptocasius montana* (Żabka, 1981) comb. nov.
15. *Ptocasius nanyuensis* (Xie & Peng, 1995) comb. nov.
16. *Ptocasius nepalica* (Żabka, 1980) comb. nov.
17. *Ptocasius nobilis* (Żabka, 1981) comb. nov.
18. *Ptocasius nova* (Żabka, 1981) comb. nov.
19. *Ptocasius orientalis* (Żabka, 1981) comb. nov.
20. *Ptocasius originalis* (Żabka, 1981) comb. nov.
21. *Ptocasius pilosa* (Żabka, 1981) comb. nov.
22. *Ptocasius pseudoflexa* (Liu, Yang & Peng, 2016) comb. nov.
23. *Ptocasius pulchella* (Li et al., 2018) comb. nov.
24. *Ptocasius senchalensis* (Prószyński, 1992) comb. nov.
25. *Ptocasius silvatica* (Żabka, 1981) comb. nov.
26. *Ptocasius simoni* (Żabka, 1981) comb. nov.
27. *Ptocasius stemmleri* (Żabka, 1981) comb. nov.
28. *Ptocasius strandi* (Żabka, 1981) comb. nov.
29. *Ptocasius supina* (Żabka, 1981) comb. nov.
30. *Ptocasius tenella* (Żabka, 1981) comb. nov.
31. *Ptocasius tenzingi* (Żabka, 1980) comb. nov.
32. *Ptocasius thakkholaica* (Żabka, 1980) comb. nov.
33. *Ptocasius thimphuica* (Żabka, 1981) comb. nov.
34. *Ptocasius urbanii* (Żabka, 1981) comb. nov.
35. *Ptocasius versicolor* (Żabka, 1981) comb. nov.
36. *Ptocasius wangdica* (Żabka, 1981) comb. nov.
37. *Ptocasius wuermli* (Żabka, 1981) comb. nov.

### *Ptocasius metzneri* sp. nov.

(Figs. 7–8, 12)

**Material**. ♂ Holotype, Prov. Prachin Buri, Sakaerat Ecol. Research Station, Thailand, [14°30′0″N, 100°52′0.12″E], No 26, evergreen forest, 01–16.06.2001, leg. E. Horváth & Gy. Sziráki, HNHM Araneae-9634. 1 ♀ Paratype, No 76, same data, HNHM Araneae-9635.

**Remark.** Despite the fact that the specimens came from different samples, they are from the same locality, and have been matched on the basis of colour pattern.

**Etymology.** The specific epithet is patronym in honour of Dr. Heiko Metzner, prominent German salticid researcher.

**Diagnosis.** In comparison with *P*. *stemmleri* tegular bump at 1 o’clock vs 2 o’clock position. In related *P*. *weyersi* the tegular bump is missing. External epigyne similar in structure to *P*. *simoni*, *P*. *supina*, *P*. *tenella*, *P*. *stemmleri*, and *P*. *helvetorum*. From *P*. *helvetorum* and *P*. *simoni* it differs by smaller copulatory openings, much longer and narrower copulatory ducts. Unlike in *P*. *stemmleri* and *P*. *supina* spermathecae much longer and multi-coiled. In opposition to *P*. *tenella* copulatory opening crevices shorter and not wavy.

**Description.** Male holotype (Figs. 7A–7I). Carapace shiny brown with dark brown eye field. Clypeus brown. Chelicerae brown, massive, with two frontal protuberances (Fig. 7F). Labium and endites dark brown, the latter apically light. Sternum dark brown. Abdomen elongate, grayish-brown. Ventral abdomen grayish, with longitudinal row of gray-brown dots. Legs dark brown. Spination of leg I: tibia ventral 2–2–2, pro- and retrolateral 1–1–1,metatarsus ventral 2–2, pro- and retrolateral 1–1. Embolus thin and long, arising from the bulb at three o’clock, and circling tegulum; seminal duct not meandering. Cymbium and tegulum each with distinctive retrolateral bumps (Figs. 7G–7I). Retrolateral tibial apophysis pointed reaching the base of embolus. Dimensions: CL 3.40, CW 2.60, CH 12.10, EFL 2.50, AEW 1.90, PEW 2.20, AL 3.60, AW 2.05.

Female paratype (Figs. 8A–8H). Carapace shiny brown with brown eye field and black eye surroundings. Clypeus brown with sparse white hairs. Chelicerae brown, labium and endites brown, the latter apically light. Sternum light brown. Abdomen grayish-brown. With contrasting wide transverse stripes. Ventral abdomen white, with longitudinal rows of light-brown dots. Legs yellowish with darker femora. Spination of leg I: tibia ventral 2–2–2, prolateral 1–1, metatarsus ventral 2–2. Epigynal plate almost round, with two pockets more distant from each other than to the copulatory openings. Copulatory openings well sclerotized oriented towards each other. Copulatory ducts C-shaped, long, rather narrow. Spermathecae long, multi-coiled, the terminal chambers pear-shaped. Dimensions: CL 2.00, CW 1.70, CH 1.55, EFL 1.30, AEW 1.50, PEW 1.35, AL 3.30, AW 2.60.

**Distribution**. Known only from type locality in Thailand (Fig. 12).

### *Ptocasius sakaerat* sp. nov.

(Figs. 9 and 12)

**Material**. ♀ Holotype, Prov. Prachin Buri, Sakaerat Ecol. Research Station, Thailand, [14°30′0″N, 100°52′0.12″E], No 96, evergreen forest, 01–16.06.2001, leg. E. Horváth & Gy. Sziráki, HNHM Araneae-9636. Paratypes: 2 ♀ ♀, 2 juv. No 72, same data, HNHM Araneae-9637.

**Etymology.** The specific epithet is derived from the name of the Environmental Research Station: Sakaerat, where the type specimens were found.

**Diagnosis.** Similarly as in *P*. *bilaguncula, P*. *montana, P*. *nanyuensis, P*. *nobilis, P*. *pulchella, P*. *senchalensis* and *P*. *wuermli*, epigynal pockets close to each other, but copulatory ducts longer and narrower, spermathecae more complex and closer the epigastric furrow.

**Description.** Female holotype (Figs. 9A–9H). Carapace shiny brown with dark brown eye field. Clypeus brown, chelicerae, labium and endites dark brown, the latter apically light. Sternum dark brown. Abdomen elongate, grayish-brown with chevron-like pattern and light stripe across the middle (Fig. 9A). Ventral abdomen grayish, with longitudinal rows of dots. Legs dark brown. Spination of leg I: tibia ventral 2–2–2, prolateral 1–1,metatarsus ventral 2–2. Epigynal plate ovoid, with two posterior central pockets very close to each other, almost merging. Copulatory openings well sclerotized, creviced, oriented diagonally to each other. Copulatory ducts relatively narrow and long, spermathecae long and multi-looped. Dimensions: CL 2.55, CW 2.20, CH 1.55, EFL 1.20, AEW 1.80, PEW 2.00, AL 4.10, AW 2.60.

**Distribution**. Known only from the type locality in Thailand (Fig. 12).

## Conclusions

1. Five new salticid species are described, increasing the number of species recorded from Thailand to 51.

2. Thirty seven species of *Yaginumaella* are transferred to *Ptocasius*.

3. The Himalayan biodiversity hotspot for *Ptocasius* is discussed in reference to bioclimatic and geological history.

##  Supplemental Information

10.7717/peerj.9352/supp-1 File S1 Distribution records of Epeus and Ptocasius Raw data for Figures 11 and 13.

10.7717/peerj.9352/supp-2 File S2 Specimens location and accession numbers

10.7717/peerj.9352/supp-3 Supplemental Information 3 PRISMA checklist

## Abbreviations used in the text and figure legends

AEW: anterior eye width
ag: accessory gland
AME: anterior median eyes
AL: abdomen length
AW: abdomen width
ca: cymbial apophysis
cd: copulatory duct
CH: cephalothorax height
CL: cephalothorax length
co: copulatory opening
CW: cephalothorax width
EFL: eye field length
e: embolus
fd: fertilisation duct
o: outgrowth
p: pocket
PEW: posterior eye width
PLE: posterior lateral eyes
PME: posterior medial eyes
pr: protuberance
psl: proximal spermathecal loops
rta: retrolateral tibial apophysis
s: spermatheca
sd: sperm duct
tb: tegular bump
tl: tegular lobe

## Additional Information and Declarations

### Data Availability

The following information was supplied regarding data availability: Distribution records of Epeus and Ptocasius are available in File S1. Described specimens with accession numbers are available in File S2; the specimens are located at the Hungarian Natural History Museum, Budapest, Hungary; RMNH Naturalis biodiversity centre in Leiden, Nederlands.

### New Species Registration

The following information was supplied regarding the registration of a newly described species: Publication LSID: urn:lsid:zoobank.org:pub:0B55634A-1D0D-49BD-BE23- 669C3630715C Salticidae LSID: urn:lsid:zoobank.org:act:174BFBAF-7027-4BC5-B872-31B2D0698A9A Epeus Peckham & Peckham 1886 LSID: urn:lsid:zoobank.org:act:61F57057-F03D-4EFD-BCDC-C16BC0B87EEB Epeus daiqini sp. nov. LSID: urn:lsid:zoobank.org:act:B34BAE44-E8D2-4E26-8289-00E1AE015571 Epeus pallidus sp. nov. LSID: urn:lsid:zoobank.org:act:E9770CAD-142D-47BF-A830-7C36A24D4447 Epeus szirakii sp. nov. LSID: urn:lsid:zoobank.org:act:BBE585D5-B67E-49B6-AA4F-33F7F93A9254 Ptocasius LSID: urn:lsid:zoobank.org:act:319E57CE-D79D-4C6A-9155-596356D69539 Ptocasius metzneri sp. nov. LSID: urn:lsid:zoobank.org:act:B5B405DB-0B44-4200-9D94-0D9FBE8BCA1B Ptocasius sakaerat sp. nov. LSID: nov. urn:lsid:zoobank.org:act:0FD99080-CE3E-4C83-AA16-ECF155F6EBB9.
