# Supplementary material for: Salticidae (Arachnida, Araneae) of Thailand: new species and records of Epeus Peckham & Peckham, 1886 and Ptocasius Simon, 1885
Source: PeerJ. 2020 Jun 22;8:e9352. doi: 10.7717/peerj.9352 (PMC7316081; doi:10.7717/peerj.9352)
Supplement: File S2 [file peerj-08-9352-s002.docx]

Material examined with specimen numbers and the name of repository where each specimen is deposited:

| specimen | specimen number | repository |
| --- | --- | --- |
| ***Epeus tener* (Simon, 1877)** 1♂, | HNHM Araneae-9630 | Hungarian Natural History Museum, Budapest, Hungary |
| ***Epeus daiqini* sp. nov.,**  Holotype 1 ♂ | RNMH.ARA.18409 | RMNH Naturalis biodiversity centre in Leiden, Nederlands |
| ***Epeus daiqini* sp. nov.,**  Paratypes: 3♀♀, 3 juv. | RNMH.ARA.18410 | RMNH Naturalis biodiversity centre in Leiden, Nederlands |
| ***Epeus pallidus* sp. nov.,**  Holotype 1 ♀ | HNHM Araneae-9631 | Hungarian Natural History Museum, Budapest, Hungary |
| ***Epeus pallidus* sp. nov.,**  Paratype: 1♀ | HNHM Araneae-9632 | Hungarian Natural History Museum, Budapest, Hungary |
| ***Epeus szirakii* sp. nov.**  Holotype 1♀ | HNHM Araneae-9633 | Hungarian Natural History Museum, Budapest, Hungary |
| ***Ptocasius metzneri* sp. nov.**  1♂ Holotype | HNHM Araneae-9634 | Hungarian Natural History Museum, Budapest, Hungary |
| ***Ptocasius metzneri* sp. nov.** 1♀ Paratype | HNHM Araneae-9635 | Hungarian Natural History Museum, Budapest, Hungary |
| ***Ptocasius sakaerat* sp. nov.** ♀ Holotype | HNHM Araneae-9636 | Hungarian Natural History Museum, Budapest, Hungary |
| ***Ptocasius sakaerat* sp. nov.** Paratypes: 2♀♀, 2 juv. | HNHM Araneae-9637 | Hungarian Natural History Museum, Budapest, Hungary |
