# Supplementary material for: Salticidae (Arachnida, Araneae) of Thailand: new species and records of Epeus Peckham & Peckham, 1886 and Ptocasius Simon, 1885
Source: PeerJ. 2020 Jun 22;8:e9352. doi: 10.7717/peerj.9352 (PMC7316081; doi:10.7717/peerj.9352)
Supplement: Supplemental Information 3 [file peerj-08-9352-s003.doc]

| **Section/topic** | **#** | **Checklist item** | **Reported on page #** |
| --- | --- | --- | --- |
| **TITLE** | | |  |
| Title | 1 | Identify the report as a systematic review: “ Salticidae (Arachnida, Araneae) of Thailand. New species and records of *Epeus* Peckham & Peckham, 1886 and *Ptocasius* Simon, 1885” | 1 |
| **ABSTRACT** | | |  |
| Structured summary | 2 | Three new species of *Epeus* Peckham & Peckham, 1886, *Epeus daiqini* (♂♀), *Epeus pallidus* (♀), *Epeus szirakii* (♀) and two new once of *Ptacasius* Simon, 1885, *Ptocasius metzneri* (♂♀) and *Ptocasius sakaerat* (♀) are describe from Thailand. The documentation of *E. tener* is completed. The genus *Ptocasius* is newly defined due to inclusion of 37 species previously described in *Yaginumaella* Prószyński, 1979. Relationships and distribution of both genera are discussed. | 2 |
| **INTRODUCTION** | | |  |
| Rationale | 3 | All species are new for science and Ptocasius is a new generic record for Thailand, which seems to be a suffusion justification to publish the data. | 2 |
| Objectives | 4 | As the result of the study we have described five new species, propose a new synonyms for 37 species, and analysed the distribution of both genera. | 2 |
| **METHODS** | | |  |
| Protocol and registration | 5 | Standard protocol for taxonomic study has been applied. Methods of study included morphological analyses of data (body and genitalic characters and measurents). The ZooBank LSIDs. | 3 |
| Eligibility criteria | 6 | The adult specimens were randomly collected in different biota and microhabitats, and they were assumed to represent typical characters for the taxa. | 3 |
| Information sources | 7 | WSC and other literature sources, information provided by the collectors, and the data provided by the authors of photographs (J. Koch, M. Bartos and D.J. Court). | 3 |
| Search | 8 | The only data base we used is WSC. The paper is not quantitative. | 3 |
| Study selection | 9 | We are experienced Salticid taxonomist with a profound knowledge of the group. |  |
| Data collection process | 10 | The process of collecting data was very simple: we have just got the specimens from the collectors, obtained information of localities and conditions, selected necessary literature, compared and discussed the data. No “sopfisticated “ analises were necessary for this kind of paper. | 3 |
| Data items | 11 | We included all variable specimens for study. | 3 |
| Risk of bias in individual studies | 12 | Not applicable to this kind of paper. |  |
| Summary measures | 13 | Not for this kind of paper. |  |
| Synthesis of results | 14 | Not for this kind of paper. |  |

Page 1 of 2

| **Section/topic** | **#** | **Checklist item** | **Reported on page #** |
| --- | --- | --- | --- |
| Risk of bias across studies | 15 | No risk of bias. |  |
| Additional analyses | 16 | No additional methods. |  |
| **RESULTS** | | |  |
| Study selection | 17 | Not applicable. |  |
| Study characteristics | 18 | The material for study is given in the text, the comparison data are taken from the papers listed in references. | 7-9, 13-15 |
| Risk of bias within studies | 19 | No risk. |  |
| Results of individual studies | 20 | The main result is discovery of five new species, which enriches our knowledge of Salticidae. | 5-16 |
| Synthesis of results | 21 | No meta analysies in the text. |  |
| Risk of bias across studies | 22 | No risk. |  |
| Additional analysis | 23 | No additional analyses. |  |
| **DISCUSSION** | | |  |
| Summary of evidence | 24 | Description of five new species distinguished on the basis of authors’ knowledge of the group and literature. | 5-16 |
| Limitations | 25 | No limitations, no risk of bias. |  |
| Conclusions | 26 | We have provided the data which could be used in taxonomic and biodiversity research. | 6-7, 13-14 |
| **FUNDING** | | |  |
| Funding | 27 | Siedlce University of Natural Sciences and Humanities, project 18/91/S |  |

*From:*  Moher D, Liberati A, Tetzlaff J, Altman DG, The PRISMA Group (2009). Preferred Reporting Items for Systematic Reviews and Meta-Analyses: The PRISMA Statement. PLoS Med 6(7): e1000097. doi:10.1371/journal.pmed1000097

For more information, visit: **www.prisma-statement.org**.

Page 2 of 2
